# Validation of the Oxford classification of IgA nephropathy for pediatric patients from China

**DOI:** 10.1186/1471-2369-13-158

**Published:** 2012-11-27

**Authors:** Weibo Le, Cai-Hong Zeng, Zhangsuo Liu, Dong Liu, Qing Yang, Rui-Xia Lin, Zheng-Kun Xia, Zhong-Min Fan, Guanghua Zhu, Ying Wu, Hong Xu, Yihui Zhai, Ying Ding, Xiaoqing Yang, Shaoshan Liang, Hao Chen, Feng Xu, Qian Huang, Hongbing Shen, Jianming Wang, Agnes B Fogo, Zhi-Hong Liu

**Affiliations:** 1 Research Institute of Nephrology, Jinling Hospital, Nanjing University School of Medicine, Nanjing, China; 2 Department of Nephrology, the First Affiliated Hospital of Zhengzhou University, Zhengzhou, China; 3 Department of Nephrology, Yuying Children’s Hospital Affiliated to Wenzhou Medical College, Wenzhou, Zhejiang, China; 4 Department of Pediatrics, Jinling Hospital, Nanjing University School of Medicine, Nanjing, China; 5 Department of Nephrology and Rheumatology, Children’s Hospital of Shanghai Jiaotong University, Shanghai, China; 6 Department of Nephrology and Rheumatology, Children’s Hospital of Fudan University, Shanghai, China; 7 Department of Pediatrics, The first affiliated hospital of henan college of TCM, Zhengzhou, China; 8 Department of Epidemiology and Biostatistics & Ministry of Education Key Lab for Modern Toxicology, School of Public Health, Nanjing Medical University, Nanjing, China; 9 Department of Pathology, Microbiology and Immunology, Vanderbilt University Medical Center, Nashville, TN, USA

**Keywords:** Glomerulonephritis, IgA nephropathy, Oxford classification, Children, Pediatrics

## Abstract

**Background:**

The Oxford classification of IgA nephropathy (IgAN) provides a useful tool for prediction of renal prognosis. However, the application of this classification in children with IgAN needs validation in different patient populations.

**Methods:**

A total of 218 children with IgAN from 7 renal centers in China were enrolled. The inclusion criteria was similar to the original Oxford study.

**Results:**

There were 98 patients (45%) with mesangial proliferation (M1), 51 patients (23%) with endocapillary proliferation (E1), 136 patients (62%) with segmental sclerosis/adhesion lesion (S1), 13 patients (6%) with moderate tubulointerstitial fibrosis (T1 26-50% of cortex scarred), and only 2 patients (1%) with severe tubulointerstitial fibrosis (T2, >50% of cortex scarred). During a median follow-up duration of 56 months, 24 children (12.4%) developed ESRD or 50% decline in renal function. In univariate COX analysis, we found that tubular atrophy/interstitial fibrosis (HR 4.3, 95%CI 1.8-10.5, P < 0.001) and segmental glomerulosclerosis (HR 9.2 1.2-68.6, P = 0.03) were significant predictors of renal outcome. However, mesangial hypercellularity, endocapillary proliferation, crescents, and necrosis were not associated with renal prognosis. In the multivariate COX regression model, none of these pathologic lesions were shown to be independent risk factors of unfavorable renal outcome except for tubular atrophy/interstitial fibrosis (HR 2.9, 95%CI 1.0-7.9 P = 0.04).

**Conclusions:**

We confirmed tubular atrophy/interstitial fibrosis was the only feature independently associated with renal outcomes in Chinese children with IgAN.

## Background

IgA nephropathy (IgAN) is the most common primary glomerulonephritis worldwide. Patients with IgAN have variable clinical courses, and the decision on which patients to treat should be based on prognostic factors and the risk of progression. Although estimation of the prognosis has mainly been based on clinical characteristics, pathological features have also been reported as risk factors for progression [1,2]. Several histologic classification systems have been devised for predicting progression of IgAN [3-7]. However, none has become widely used [8], partly because the reproducibility of histological variables were not tested in those classifications.

The new Oxford classification of IgAN, based on 265 patients collected from eight countries on four continents, identified four definitive histological features, with high reproducibility and low collinearity, for the prediction of renal prognosis of IgAN: mesangial hypercellularity (M), endocapillary proliferation (E), segmental sclerosis or adhesion (S), and tubular atrophy/interstitial fibrosis (T) [8,9]. However, the development of the Oxford classification involved patients with an age range encompassing pediatric and adult patients. Several studies [10-14], including the original Oxford study, have shown that the histological features of IgAN in children and adults are remarkably different. Compared with adults, children with IgAN showed significantly more mesangial and endocapillary hypercellularity, and less chronic tubulointerstitial and vascular damage [10-14]. Hence, whether the classification system has the similar predictive power for children with IgAN in different populations needs to be validated further.

Recently, several validation studies of the Oxford classification have been published [15-20], however, most of these studies focused on adult patients with IgAN. A study performed by Edström et. al [17] found that the presence of S was not associated with the long-term renal outcome in a cohort of pediatric IgAN patients from Sweden. Shima et. al [19] analyzed 161 consecutive children with IgAN from Japan and found that M, T, and crescents (>30%) were significant univariate analyses. We herein report a multicenter validation study of the Oxford classification, using similar inclusion criteria and statistical analysis, in a cohort of children with IgAN from China.

## Methods

### Inclusion criteria and clinical data set

Cases were biopsy-proven IgAN with age <18 years old, and an initial eGFR ≥30 ml/min per 1.73m^2^, and initial proteinuria ≥0.5g per 24 h, and total number of glomeruli ≥10 for analysis. Cases that were followed up ≥12 months, and those that had progressed to ESRD, regardless of the duration of follow-up, were included. Cases with secondary causes of mesangial IgA deposits such as Henoch–Schönlein purpura or those with comorbid conditions such as diabetes mellitus, were excluded.

Demographic data included gender, ethnicity, date of birth, date of initial presenting clinical features, and age at biopsy. Clinical parameters collected within one month of date of biopsy and during follow-up included systolic and diastolic blood pressure, weight, height, serum creatinine, albumin, cholesterol, triglyceride, and 24h urine protein or urine protein:creatinine ratio, count of urine red blood cells, and macroscopic hematuria. Treatment modalities were recorded including immunosuppressive agents, statins, tonsillectomy, and a number of antihypertensive medications.

### Definitions

Pathology definitions used were the same as in the original Oxford Classification [9]. eGFR was estimated using the Schwartz formula; in patients aged >16 years at the time of biopsy, only the MDRD equation was used. ESRD was defined as eGFR < 15 ml/min per 1.73m^2^. A combined event was defined as ESRD or 50% reduction in initial eGFR. MAP was defined as diastolic pressure plus 1/3 pulse pressure. For each patient, an average MAP and proteinuria were determined for each year of observation. Time-average MAP and proteinuria represent the average of these annual values. Immunosuppressive treatment is reported as type and the duration of therapy. RAS blockade included any exposure to either angiotensin converting enzyme inhibitor or angiotensin receptor blocker, or both.

### Histological scoring

The exact biopsy tissue sections to be scored were marked on the PAS slides. The scoring was done by two pathologists (CH Z and SS L). The scoring sheet was based on the Oxford classification of IgAN, and eight pathological variables, namely mesangial hypercellularity (M), endocapillary proliferation (E), segmental sclerosis or adhesion (S), crescents (C), glomerulus necrosis (N), tubular atrophy/interstitial fibrosis (T), artery score, and malignant vascular changes were assessed. Capillary necrosis was scored from all the slides of each case, including H&E, PAS, PASM and Masson trichrome staining, so that this very segmental lesion would be more likely to be identified.

### Statistical analysis

Normally distributed variables were expressed as mean ± S.D. and differences among groups were analyzed by Student t-test or one-way ANOVA. Qualitative data were described as percentages and analyzed using Chi-square (χ^2^) test. Non-parametric variables were expressed as median, and compared using either Mann–Whitney or Kruskal–Wallis test.

The renal survival, estimated by a 50% reduction in renal function or ESRD (the combined event) was used as the primary outcome. Renal survival curves related to pathological variables were calculated using the Kaplan–Meier method, and comparisons were made with a log-rank test. Cox regression was used to determine predictors of renal outcome. The P-value reported was two-sided and a value of less than 0.05 was considered statistically significant. CIs included 95% of predicted values. All analyses were performed using R (Version 2.131).

The protocol followed in the present study was approved by the Jinling Hospital Ethics Committee on Human Experimentation(NO. 2010-NLY-024). Due to the retrospective nature of the study, written informed consent for participation in the study was waived .

## Results

### Clinical and pathological characteristics

A total of 218 pediatric patients were recruited from 7 different renal centers in China. Clinical features at biopsy and during follow-up are shown in Table 1. At the time of renal biopsy, the median age was 14 years, with male (65%) predominance. Median proteinuria was 1.5 g/d. During a median follow-up duration of 56 months, 24 children (12.4%) developed ESRD or 50% decline in renal function. In general, the clinical characteristics in this cohort were very similar to the pediatric patients in the original Oxford cohort. Compared with 161 children from Japan, reported by Shima et al. [19], children in this cohort and the original Oxford cohort had more severe proteinuria at biopsy, and were treated with more RASB and immunosuppressive therapy during follow-up. Compared with 1026 adults in another multi-center validation cohort from China [21], children in this cohort were also more likely to have a history of macroscopic hematuria, to have higher initial eGFR, and to receive more immunosuppressive therapy but less antihypertensive therapy.

**Table 1:** Clinical characteristics at the time of biopsy and follow-up in 218 pediatric patients with IgA nephropathy

	**Current study**	**Oxford study** (**Children**)	**Shima et al**. [19]
***At time of biopsy***	n = 218	n = 59	n = 161
Age (years)	14 (2–17.9)	13 (4–17.9)	11.7(3.6-19.4)
Female	35%	25%	37%
Hypertensive before biopsy	6.5%	NA	NA
MAP (mm Hg)	88 ± 11	84 ± 10	79 ± 11
eGFR (ml/min per 1.73 m^2^)	134 ± 42	120 ± 43	103 ± 30
Proteinuria (g/day)	1.5(0.5-8.0)	2 (0.5-7.8)	0.7 (0.0–13.7)
Previous macroscopic hematuria	57%	60%	66%
***Follow***-***up***			
Duration of follow-up (months)	56(12–182)	62 (20–268)	54 (12–170)
50% decline in eGFR or ESRD	12.4%	NA	NA
MAP (mm Hg)	86 ± 10	86 ± 8	NA
Proteinuria (g/day)	0.6 (0.1-4.9)	0.9 (0.1–7.0)	NA
Immunosuppression	56%	48%	16%
Prednisone	51%	48%	16%
Others	28.6%	17%	11%
Treated with RASB	61.5%	56%	NA

Abbreviations: RASB, renin-angiotensin system blockade; eGFR, estimated glomerular filtration rate; MAP, mean arterial pressure; ESRD, end stage renal disease; NA, not available.

Values are expressed as mean ± s.d. or median (range). Calculation of MAP, eGFR, and proteinuria is detailed in the text.

**Figure 1:**
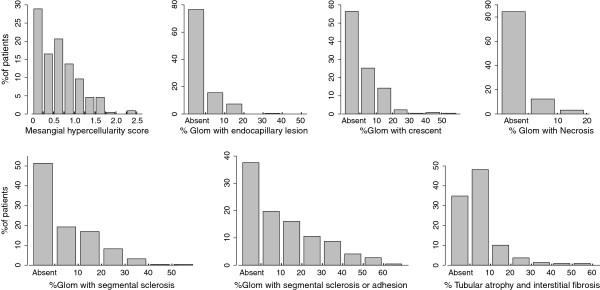
**Distribution of pathological features.** Percentage of patients with each pathological feature.

There were a median of 21 glomeruli per biopsy (interquartile range 16–29). Distribution of several pathology findings is shown in Figure 1. There were 98 patients (45%) with mesangial proliferation (M1), 51 patients (23%) with endocapillary proliferation (E1), 136 patients (62%) with segmental sclerosis/adhesion lesion (S1), 13 patients (6%) with moderate tubulointerstitial fibrosis (T1, 26-50% of cortex scarred), and only 2 patients (1%) with severe tubulointerstitial fibrosis (T2, >50% of cortex scarred). As T2 was seen in only two cases in the current study, we merged T1 and T2 together in the following analyses. Crescents were seen in 95 cases (44%), however, the median ratio of glomeruli with crescents was 9%, and only one patients showed crescents involving greater than 50% of glomeruli. Capillary necrosis was seen in 34 cases (16%).

### Clinicopathological correlations at the time of biopsy

The clinicopathological correlations at the time of biopsy are shown in Table 2. The M, E, S, T scores, and crescents were strongly associated with proteinuria at biopsy, and the M, S, and T scores were strongly associated with MAP at biopsy. None of the lesions were significantly associated with eGFR at the time of biopsy except for tubular atrophy/interstitial fibrosis. Children with T1 or T2 had a significantly lower eGFR compared with those without (P =0.004). The E lesion and capillary necrosis were not correlated with any of the clinical features.

**Table 2:** Relations between clinical and histological variables at time of biopsy

	**MAP**	**eGFR**	**Proteinuria**
Mesangial hypercellularity score (M)			
≤0.5	87 ± 11	133 ± 43	1.2 (0.81-2.2)
>0.5	90 ± 11	135 ± 40	1.9 (1.1-2.9)
P-value	0.03	0.8	0.002
Endocapillary proliferation (E)			
Absent	87 ± 11	135 ± 42	1.3 (0.86-2.3)
Present	90 ± 12	130 ± 43	2.1 (1.1-3.0)
P-value	0.7	0.4	0.003
Segmental glomerulosclerosis (S-alone)			
Absent	85 ± 10	135 ± 43	1.2 (0.78-2.2)
Present	92 ± 11	132 ± 41	1.7 (1.1-3.0)
P-value	<0.001	0.7	<0.001
Segmental glomerulosclerosis or adhesion (S)			
Absent	86 ± 10	138 ± 41	1.2 (0.79-2.3)
Present	90 ± 11	131 ± 42	1.6 (1.0-2.7)
P-value	<0.001	0.3	0.01
Tubular atrophy / interstitial fibrosis (T)			
T0	87 ± 11	136 ± 41	1.4 (0.89-2.5)
T1 or T2	96 ± 11	97 ± 44	2.4 (1.4-3.2)
P-value	0.009	0.004	0.03
Crescent( C)			
Absent	87 ± 11	137 ± 42	1.2 (0.8-2.2)
Present	90 ± 11	129 ± 42	1.8 (1.1-3.2)
P-value	0.09	0.2	<0.001
Glomerulus necrosis (N)			
Absent	87 ± 11	135 ± 42	1.4 (0.9-2.5)
Present	90 ± 12	125 ± 42	1.6 (0.9-2.9)
P-value	0.4	0.2	0.3

Abbreviations:eGFR, estimated glomerular filtration rate; MAP, mean arterial pressure.

Values are expressed as mean ± s.d. or median (interquartile range). Calculation of MAP, eGFR, and proteinuria is detailed in the text.

### Interaction of pathological features with therapy

The use of two major treatments, RAS blockade and immunosuppression, was assessed in relation to the selected pathological lesions (Table 3). Compared with adults in another multi-center validation cohort from China [21] and Oxford study, children have received more immunosuppressive treatment, but fewer RAS blockade. Children with M, S, T, or C received subsequent RAS blockade more often than those without those lesions. Those with crescent were more likely to receive immunosuppressive treatment than those without crescents. However, children with E were likely to have an equal chance to receive immunosuppressive treatment (P = 0.4) to those without E, as well as RAS blockade treatment (P = 0.6). There were also no significant association between the extent of E (% glomeruli with these lesions) and immunosuppression during follow up in this cohort.

**Table 3:** Therapy received during follow-up in relation to pathological features

	**%RASB (>6months)**	***P***-***value***	**% Immuno**- **Suppression**	***P***-***value***
Mesangial hypercellularity score (M)				
≤0.5	64	0.04	50	0.07
>0.5	78		64	
Endocapillary proliferation (E)				
Absent	69	0.6	54	0.4
Present	75		63	
Segmental glomerulosclerosis (S-alone)				
Absent	59	<0.001	53	0.5
Present	83		59	
Segmental glomerulosclerosis or adhesion (S)				
Absent	57	0.001	59	0.5
Present	79		54	
Tubular atrophy / interstitial fibrosis (T)				
Absent or Mild (0%-25%)	68	0.03	57%	0.8
Moderate (>25%)	100		50%	
Crescent (C)				
Absent	62	0.002	47	0.004
Present	82		68	
Glomerulus necrosis (N)				
Absent	69	0.6	55	0.7
Present	76		61	

Abbreviations: RASB, renin angiotensin system blockade.

### Correlations between pathological lesions and outcome

**Figure 2:**
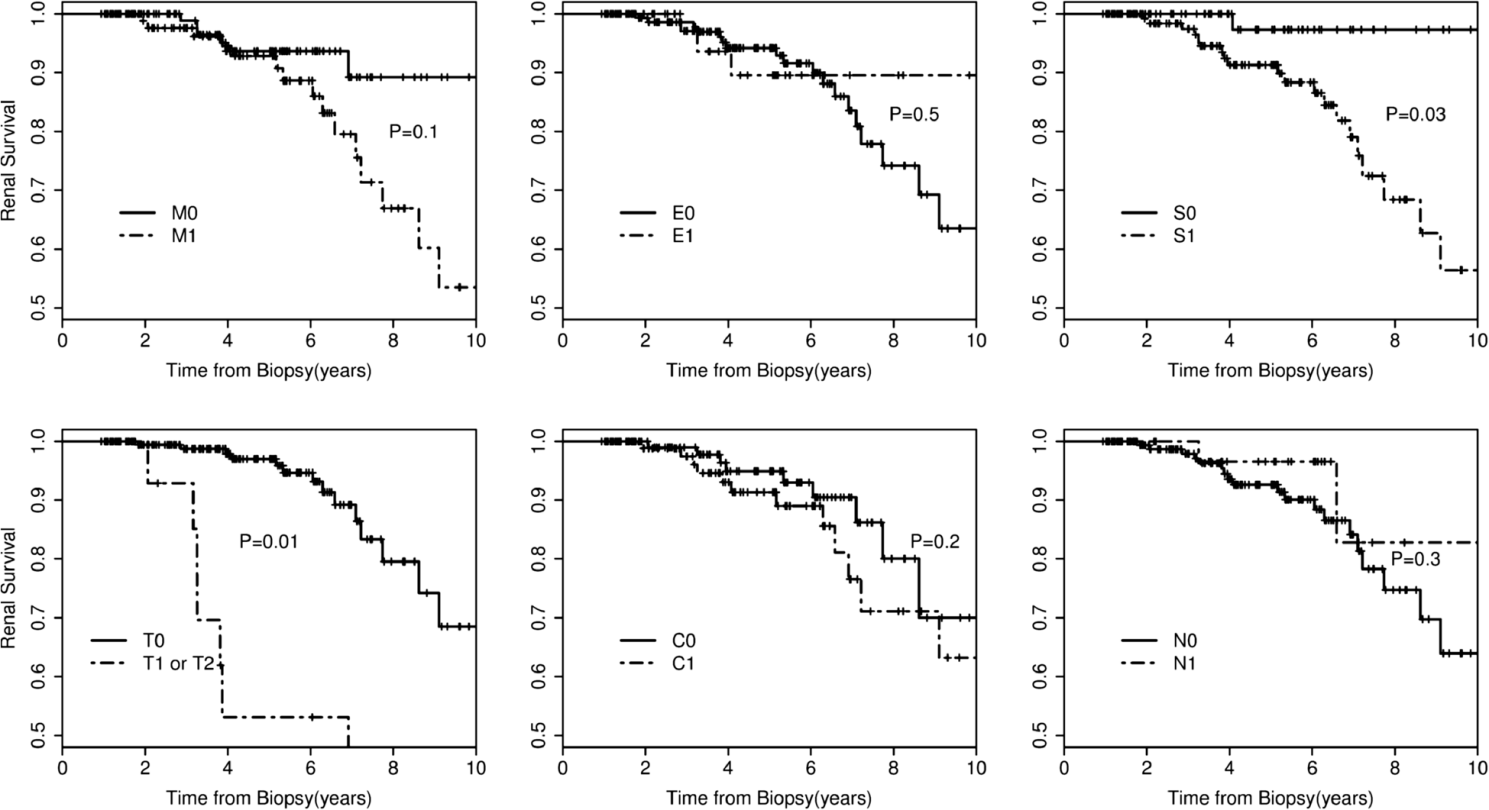
**Renal survival according to pathological variables.** M mesangial hypercellularity score, E endocapillary hypercellularity, S segmental glomerulosclerosis or adhesion, T tubular atrophy/interstitial fibrosis, C crescents, N glomerulus necrosis.

Figure 2 shows the differences in renal survival from the combined event for presence and absence of the histological findings. The Kaplan–Meier analyses showed lesion S and T were each significantly associated with renal outcome, while lesion M, C, E and necrosis were not.

The correlations between pathological lesions and renal outcome were also analyzed in a COX regression model (Table 4). The univariate COX regression model showed that lesions S (HR 9.2, 95%CI 1.2-68.6, P = 0.03) and T (HR 4.3, 95%CI 1.8-1.5, P = 0.001) were each significantly associated with renal outcome, while the lesion of M (HR 2.1, 95%CI 0.84-5.1, P = 0.1), E (HR 0.6, 95%CI 0.2-2.3, P = 0.5), C (HR 1.8, 95%CI 0.77-4.1, P = 0.2), and necrosis (HR 0.6, 95%CI 0.14-1.7, P = 0.3) were not. In the multivariate COX regression model, when adjusted for initial clinical data set (eGFR, MAP, and proteinuria), none of these pathologic lesions were shown to be independent risk factors of unfavorable renal outcome except for T (HR 2.9, 95%CI 1.0-7.9, P = 0.04).

**Table 4:** Correlations between pathological features and outcomes

	**HR (95% CI) Univariate**	***P***-***value***	**HR (95% CI) Multivariate**^**b**^	***P***-***value***
Mesangial hypercellularity score (M)				
≤0.5	1.0	0.1	1.0	0.6
>0.5	2.1 (0.84-5.1)		1.3 (0.49-3.4)	
Endocapillary proliferation (E)				
Absent	1.0	0.5	1.0	0.2
Present	0.67 (0.2-2.3)		0.44 (0.12-1.5)	
Segmental glomerulosclerosis or adhesion (S)				
Absent	1.0	0.03	1.0	0.1
Present	9.2 (1.2-68.6)		5.2 (0.6-43)	
Tubular atrophy / interstitial fibrosis (T)				
T0	1.0	0.01	1.0	0.04
T1 or T2	4.3 (1.8-10.5)		2.9 (1.0-7.9)	
Crescent (C)				
Absent	1.0	0.2		NS
Present	1.8 (0.8-4.1)			

Abbreviations: CI, confidence interval; OR, odds ratio; HR, hazard ratio. NS, not significant.

^b^Multivariate Cox regression model: multivariate with four pathological features (M,E,S,T) + initial eGFR, MAP and proteinuria.

The lesion S was defined as segmental glomerulosclerosis or adhesion in the original Oxford study. We have found that the segmental glomerulosclerosis alone (S-alone, not involving the adhesion lesions) is a more valuable pathological lesion than the defined as segmental sclerosis or adhesion lesion in 1026 adult patients from China [21]. In this study, we also evaluated the predictive value of S-alone, instead of S with or without adhesion. In univariate Cox regression analysis, children with S-alone had a 3.8-fold higher risk of renal failure than those without (95%CI: 1.3-11.1, P = 0.02). However, when adjusting the two pathology variables (lesions M and T) and the initial clinical data set (eGFR, MAP, and proteinuria), these association was not statistically significant in the multivariate Cox regression analysis (OR 2.2, 95%CI , P = 0.2).

## Discussion

The Oxford classification of IgAN provides a histopathological grading system for prediction of renal prognosis of IgAN independent of the clinical features [8,9]. However, the classification must be validated in the different cohorts of patients. This study was designed, using similar methods as in the Oxford study, to assess the validity of the new Oxford classification of IgAN in a multi-center cohort of pediatric patients from China. The clinical characteristics in our cohort were very similar to the pediatric patients in original Oxford cohort (Table 1). Our study shows that tubular interstitial fibrosis was the only pathological feature independently associated with renal outcomes in Chinese children with IgAN.

It is remarkable to notice that lesion M, E, C and N, which were all thought to be active glomerular lesions in patient with IgAN, were not independently associated with renal outcome in our study. The similar results were also showed in another validation study in 1026 Chinese adult patients [21]. Moreover, the prognostic values of M, E and C were also controversial in different validation studies [8,15-18,20,22-26]. The lesion E and C were also not statistically associated with renal outcome in the original Oxford cohorts [8]. The prognostic value of necrosis was not evaluated in the original Oxford study, as only six cases (2.3%) had this lesion in that cohort of patients. Compared with patients in the Oxford study, there were significantly more patients with necrosis (16%) in this study, however, an association between necrosis and renal outcome was not established, and similar results were also found in two other studies [24,27]. Taken together, those results indicate that there are only weak associations between present of these acute lesions (M, E, C, N) and renal outcome. Several possible explanations may account for these results. Firstly, those acute glomerular lesions only reflect the disease activity at the time of renal biopsy, and all of them are reversible after immunosuppressive treatment [28]. Secondly, the ration of glomeruli with these lesions is very important in patients with IgAN, as most of the patients have only small numbers of crescents in our study and similar finding were also showed in other studies [8,17,21]. Shima et. al [19] found that only those patients with C > 30% or E > 30% were associated with an unfavorable renal outcome in children with IgAN, indicating that the optimal cutoff ratios of these acute lesions for predicting a worse outcome should be determined in IgAN in the future. Thirdly, the inconsistent results among those validation studies may due to different inclusion criteria, as shown by Katafuchi et al. [29] and Shima et al. [19]. Finally, the lack of predictive value of this lesion may reflect inadequate statistical power, as only a small subset of patients developed ESRD or 50% decline in GFR during the follow-up in most validation studies, including the current study.

Recently, two studies about validation of the Oxford classification for pediatric IgA nephropathy were published from Japan and Sweden respectively. The most obvious difference between our study and the two previous studies is lesion S. Both Shima et. al [19] Edström et. al [17] found that present of lesion didn’t indicate a poor prognosis in IgAN. In the present study, present of lesion S were showed to be significantly associated with renal outcome in univariate COX analysis, but it failed to attain independent significance in multivariate model. A similar predictive value was shown between S and S-alone in this cohort. This may due to the different health screening practice and inclusion criteria, various treatments during follow-up, and especially the poor reproducibility (ICC) of lesion S. Children in this study have more severe proteinuria at biopsy, and received more RASB and immunosuppressive therapy during follow-up than children from Japan [19]. Given that the ICC of adhesion was poor (0.2) in the original Oxford study, the frequency distribution of S was also remarkably different among the validation studies. Taken together, these findings indicate that lesion S seems to had a weak influence on renal survival.

One of the most exciting findings in the new Oxford IgAN classification, is the question of whether this classification can predict optimal treatment for patients with IgAN. The original Oxford study showed that, in patients who received no immunosuppression, the rate of renal function decline in those with E was faster than those without, while there was no such difference in patients treated with immunosuppression. Hence, the lesion E was finally involved in the Oxford classification, and this provided indirect evidence that lesion E is assumed responsive to immunosuppressive therapy. The similar indirect evidence was also shown in a validation study from four centers in North America [16]. However, in the current cohort of patients, we do not confirm these findings. Whether lesion C, E, and N can predict optimal treatment for patients with IgAN remains unclear, and prospective clinicopathological studies are needed to investigate this possibility.

IgAN is defined as dominant or codominant staining with IgA in glomeruli by immunofluorescence or immunoperoxidase [9]. It is important to note that it may in fact simply define a group of diseases sharing identical histopathologic sequelae [30]. If that is the case, a great limitation of this histopathological classification should be recognized, for an ideal classification system should be based on pathogenic mechanisms and should thus suggest an appropriate therapeutic strategy. The classification of IgAN should also be improved based on the biomarker of pathogenic mechanisms in the future.

## Conclusions

Our study indicates tubular atrophy/interstitial fibrosis was the most powerful lesion for prediction of renal prognosis of IgAN independent of clinical features, while segmental glomerulosclerosis had a weak influence on renal survival. Mesangial hypercellularity, endocapillary hypercellularity, crescent and capillary necrosis were not associated with the renal outcome. Whether the Oxford classification can predict an optimal treatment for children of varying ethnicity with IgAN remains unclear.

## Abbreviations

IgAN: IgA nephropathy; M: Mesangial proliferation; E: Endocapillary proliferation; S: Segmental sclerosis/adhesion lesion; T: Tubulointerstitial fibrosis; C: Crescents; N: Glomerulus necrosis; S-alone: Segmental glomerulosclerosis alone; RASB: Renin-angiotensin system blockade; eGFR: Estimated glomerular filtration rate; MAP: Mean arterial pressure; ESRD: End stage renal disease.

## Competing interests

All authors declared that they have no competing interests.

## Authors’ contributions

WL and C-HZ carried out the Clinico-pathological studies, participated in the statistical analysis and drafted the manuscript. C-HZ, SL and ABF participated in the renal pathology studies and helped to draft the manuscript. ZL, DL, QY, R-xL, Z-KX, Z-MF, GZ, YW, HX, YZ, YD, XY, QH, HC, and FX participated in patients inclusion and demographic data collections. HS and JW participated in the statistical analysis. ZL conceived of the study, and participated in its design and coordination and helped to draft the manuscript. All authors read and approved the final manuscript.

## Authors’ information

Weibo Le and Cai-Hong Zeng have contributed equally to the work and are both to be considered first authors.

## Pre-publication history

The pre-publication history for this paper can be accessed here:

http://www.biomedcentral.com/1471-2369/13/158/prepub
